# The Glymphatic–Venous Axis in Brain Clearance Failure: Aquaporin-4 Dysfunction, Biomarker Imaging, and Precision Therapeutic Frontiers

**DOI:** 10.3390/ijms262110546

**Published:** 2025-10-30

**Authors:** Daniel Costea, Nicolaie Dobrin, Catalina-Ioana Tataru, Corneliu Toader, Matei Șerban, Răzvan-Adrian Covache-Busuioc, Octavian Munteanu, Ionut Bogdan Diaconescu

**Affiliations:** 1 Puls Med Association, 051885 Bucharest, Romania; costea.damiel@umft.ro (D.C.); mateiserban@innbn.com (M.Ș.); razvancovache@innbn.com (R.-A.C.-B.);; 2 Department of Neurosurgery, “Victor Babes” University of Medicine and Pharmacy, 300041 Timisoara, Romania; 3 ”Nicolae Oblu” Clinical Hospital, 700309 Iasi, Romania; 4 Clinical Department of Ophthalmology, “Carol Davila” University of Medicine and Pharmacy, 020021 Bucharest, Romania; 5 Department of Ophthalmology, Clinical Hospital for Ophthalmological Emergencies, 010464 Bucharest, Romania; 6 Department of Neurosurgery, “Carol Davila” University of Medicine and Pharmacy, 050474 Bucharest, Romania; 7 Department of Vascular Neurosurgery, National Institute of Neurology and Neurovascular Diseases, 077160 Bucharest, Romania; 8 Discipline of Anatomy Department II of Morphological Sciences, “Carol Davila” University of Medicine and Pharmacy, 050098 Bucharest, Romania

**Keywords:** glymphatic system, Aquaporin-4, brain clearance, neurodegeneration, biomarkers, venous congestion

## Abstract

The identification of brain clearance failure as a precursor to a large variety of neurodegenerative diseases has shifted fluid dynamics from a secondary to a tertiary target of brain health. The identification of the glymphatic system, detailing cerebrospinal fluid entry along perivascular spaces and exit via perivenous and meningeal lymphatic pathways, provided a challenge to previous diffusion models and established aquaporin-4–dependent astroglial polarity as a governing principle of solute transport. Multiple lines of evidence now support a coupled glymphatic–venous axis, wherein vasomotion, venous outflow, and lymphatic drainage are functionally interrelated. Failure of any axis will cascade and affect the entire axis, linking venous congestion, aquaporin-4 disassembly, and meningeal lymphatic failure to protein aggregation, neuroinflammation, edema, and intracranial hypertension. Specific lines of evidence from diffusion tensor imaging along vascular spaces, clearance MRI, and multi-omic biomarkers can provide a measure of transport. Therapeutic strategies are rapidly advancing from experimental strategies to translational approval, including behavioral optimization, closed-loop sleep stimulation, vascular and lymphatic therapies, focused ultrasound, pharmacological polarity recoupling, and regenerative bioengineering. Novel computational approaches, such as digital twin dynamic modeling and adaptive trial designs, suggest that clearance measures may serve as endpoints to be approved by the FDA. This review is intended to bridge relevant mechanistic and translational reviews, focusing on impaired clearance as an exploitable systems defect rather than an incapacitating secondary effect. Improving our understanding of the glymphatic-venous axis Injury may lead to future target strategies that advance cognitive resilience, alleviate disease burden, and improve quality of life. By clarifying the glymphatic–venous axis, we provide a mechanistic link between impaired interstitial clearance and the pathological accumulation of amyloid-β, tau, and α-synuclein in neurodegenerative diseases. The repair of aquaporin-4 polarity, venous compliance, and lymphatic drainage might therefore open new avenues for the diagnosis and treatment of Alzheimer’s and Parkinson’s disease, supplying both biomarkers of disease progression and new targets for early intervention. These translational implications not only locate clearance failure as an epiphenomenon of neurodegeneration but, more importantly, as a modifiable driver of the course of neurodegeneration.

## 1. Introduction—The Glymphatic—Venous Axis as a Systems-Level Framework for Brain Clearance

The central nervous system (CNS) generates a substantial metabolic burden, yet lacks classic lymphatic channels within its parenchyma. Thus, for over a century, physiologists have studied the means by which the brain would dispose of its metabolic waste. The earliest ideas, like the Virchow-Robin perivascular spaces, and Cushing’s fluid-dynamics ideas, hinted at specialized clearance routes, while diffusion models of solute transfer predominated for most of the twentieth century [1]. These models could never explain the rapid clearance of macromolecules observed in tracer studies, especially for peptides like amyloid-β and tau [2]. With the discovery of the glymphatic system in 2012, the problem was shifted to a perivascular convective pathway driven by influx of cerebrospinal fluid (CSF) into periarterial spaces and efflux in perivenous paths, through aquaporin-4 (AQP4) channels on astrocytic endfeet [3]. Nonetheless, despite the adoption of such concepts, several issues remained. How are these flows initiated and sustained, and where exactly are they exiting the intracranial compartment?

Over the last 2 years, a number of original studies have refined these questions and begun to arrive at mechanistic answers. Multiple lines of evidence now suggest that the glymphatic system must be reconciled with venous hemodynamics and meningeal lymphatics [4]. This integrative systems approach, which we will thus refer to in the following as the glymphatic-venous axis, relates state-dependent oscillations of arterial and brain tissue dynamics to venous outflow and meningeal lymphatic drainage. What separates this axis from others is that it is a convergence clearance pathway: not just a linear sequential pathway, but a biomechanically coupled pathway in which failure at one limb transfers to the whole system dysfunction [5].

### 1.1. State-Dependent Vasomotion and Neural Oscillations as Proximal Drivers of Influx

While prior models have proposed cardiac and respiratory pulsatility as potential inputs for perivascular transport, more recent work in vivo using fiber photometry, opto-genetics, and multi-echo fMRI has shown that slow vasomotion, starting frequencies < 0.1 Hz, is the true driver for glymphatic influx [6]. They are robustly regulated by the locus coeruleus, which releases norepinephrine, suppressing vasomotion during wakefulness and promoting vasomotion during non-REM sleep. Optogenetic silencing of nora-drenergic neurons depletes glymphatic transport, while pharmacological manipulation that promotes vasomotion increases transport [7].

The second, but critical point is that neural activity alters vascular oscillations. First, entrainment of cortical networks to gamma ranges (~40 Hz) has been shown to modulate clearance, in part, due to the coupling of vascular and neural rhythms, which induces astrocytic reactivity [8]. Together, these imply a nested, integrated control system whereby slow vasomotion provides the foundation for convective force/drive, while the often much faster neural oscillations are the loci of top-down modulation of glymphatic transport. So, rather than viewing sleep as a yes-or-no condition of transport, these results suggest that at least some stage of sleep and the electro-chemical milieu determine transport efficacy. This is critical for translation limitations as sedatives/hypnotics could potentially each interfere or allow for these oscillatory regimes [9].

### 1.2. Anatomical Organization of Efflux and Venous Integration

For most of the last 10 years, the drainage side of glymphatic flow was thought to be completely non-descript. The long-held belief that solutes exit parenchyma to subarachnoid CSF diffusively has been challenged by newly reported human imaging and two-photon dissection high-resolution data. In 2024, contrast-enhanced MRI and histological testing identified meningeal lymphatic vessels (mLVs) positioned next to dural venous sinuses and meningeal arteries. mLVs have been shown to express canonical lymphatic endothelial markers (LYVE-1, Prox1, podoplanin), and this is established definitively, thus confirming them as true lymphatic vessels. Their position immediately next to venous sinuses allows for a route of mLVs to connect perivenous efflux with extracranial lymphatic drainage [10,11].

Additionally, perineural routes along cranial nerves, specifically the olfactory pathway, can be co-opted as alternate pathways for efflux of CSF or solutes. Imaging comparison studies suggest different MRI sequences preferentially engage various outflow pathways, thus demonstrating that clearance is heterogeneous. Thus, the efflux properties of parenchyma are not homogenous but represented across multiple structurally specialized pathways, and each pathway is differentially controlled by venous and meningeal anatomy. These observations are preliminary evidence toward the proposal that venous territories are not passive storage endpoints, but rather part of an organized system of clearance [12].

### 1.3. Venous Hemodynamics as a Bottleneck and Regulator

Recent groundbreaking information learned in 2024–2025 was regarding the regulation of venous hemodynamics. In patient-specific simulations of idiopathic intracranial hypertension (IIH) with transverse sinus stenosis, we found that stenting not only dramatically altered venous flow and wall shear stress, but it also had a meaningful symptomatic complication, and in exploratory imaging, normalization of CSF distribution patterns suggestive of improved clearance [13]. At the same time, preclinical experiments suggested that experimentally altering venous pressures altered tracer drainage into dural lymphatics when obstructed but restored patency when it was re-established. These findings highlight venous hemodynamics as the dominant rate-limiting factor along the clearance axis. Whereas arteries exhibit associated pulsatility, veins are impeded by compliance and threshold pressures, which can result in severely impaired efflux. Glymphatic inflow and venous outflow must be considered two halves of one system in the physiological sense. Venous drainage deviances can occur upstream and constrict parenchymal clearance, independent of arterial flow [14].

### 1.4. Quantitative Biomarkers and Reproducibility of Clearance Assessment

Translating the glymphatic-venous axis to clinical paradigms and the potential to measure in a repeatable and reproducible way. The 2024 CHAMONIX study provided multi-center reproducibility estimates of the [-DDI-ALPS index, a diffusion MRI metric that measures, in essence, the results of perivascular transport difficulties [15,16]. The study provided confidence intervals and minimally detectable variations, enabling the authors to quantify differences across scanners and protocols and laid the groundwork for transitioning to longitudinal studies. Additional work from studies examined with populations, notably alcohol use disorder populations, offered some external validity- including similar findings to cognitive metrics that have also been validated in those studies [17].

Not only can diffusion MRI be used to characterize the glymphatic axis, but simple FLAIR imaging can also detect artifactual signal changes that are dependent on pulsatility and CSF flow, and correlate with glymphatic functional and throughput metrics. The 2025 study explored a specific hemipraxium of pulsatility signatures and their physiological correlates, which offer another low-hanging fruit to transition clearance measures into clinical practice [18]. Another feasibility study confirmed that machine learning can optimize the detection of perivascular spaces using deep learning algorithms that can standardize structural quantitation from anomalous data. These technological advancements will all help the glymphatic-venous axis transition from a description-based hypothesis to a measurable physiological apparatus [19].

### 1.5. Systems Biomechanics and Integrative Modeling

New computational models have advanced clearance physiology into the territory of systems biomechanics. Seven-compartment porous-media simulations, including subject-specific geometries and intracranial pressures, can emulate the infusion-test dynamics and quantify glymphatic flow under different simple conditions. These models indicate that intracranial pressure, tissue compliance, and perivascular geometry jointly determine the efficiency of clearance [20].

Other conceptual modeling has illustrated the mechanism of brain tissue pulsations, independent of arterial wall motion, to augment CSF displacement through perivascular spaces, paralleling direct observations of parenchymal displacement during sleep, and adding to the notion that brain-wide biomechanical states influence clearance, not just vascular input. Such modeling provides a physical and rational reason venous obstruction—via compliance and boundary condition alteration—may transmit dysfunction upstream into the glymphatic inflow [21].

### 1.6. Toward a Working Definition and Implications

From these conceptual models, we may tentatively define the glymphatic–venous axis as a dependent-state biologically couched clearance system consisting of:

1. Periarterial CSF influx into parenchyma driven by slow vasomotion, neural oscillatory states, and brain tissue pulsations [22];
2. Parenchymal interstitial transport mediated by astrocytic AQP4 regulates neurovascular coupling and matrix composition [23];
3. Perivenous efflux drains through the dural venous regions and meningeal lymphatics, which are limited by venous compliance and resistance [24].

This definition generates testable hypotheses:

- Any intolerable combinations on this system that inhibit slow flow (e.g., inadequate sleep hygiene, sedatives) will inhibit inflow and correspond to low glymphatic indices;
- Venous stenosis (increased vascular wall stiffness) or hypertension will inhibit efflux hunt as manifested by increased perivascular space diffusion or slow clearance rate of tracers;
- Restoring venous outflow (stenting) or boosting sleep oscillations (neuromodulation or circadian rhythms) will result in restoring clinical measures of clearance ability.

### 1.7. Scope and Novelty of This Review

The goal of integrating arterial driving, parenchymal coupling, venous regulation, and lymphatic drainage into a single cohesive framework is to promote the notion of brain clearance as a true functional physiological system. Previous reviews have typically focused on one of these elements well, whether it is glymphatic inflow, venous compliance, or meningeal lymphatics; however, the relationship between these elements has remained poorly defined. This synthesis represents a modest step toward integrating these mechanisms into a single dynamic concept, which could be described as a glymphatic–venous axis, in which each element functions cooperatively and interactively with the others.

What gives novelty to this approach is not the ambition to replace existing mechanisms, but the desire to integrate established findings at different scales, ranging from astrocytic AQP4 polarity and the biophysics of the extracellular matrix to the boundary conditions governing the venous compartments and the contractile nature of the lymphatics. By exploring clearance mechanisms as a coupled biomechanical feedback loop rather than a linear process, it becomes possible to educate a variety of disease processes in a shared physiological language.

An integrative approach of this nature is still in its infancy and should be approached with caution. Its justification lies in the attempt to organize the current evidence into a system-level approach, which can be plotted like a map, allowing direction to be established for future studies rather than attempting to provide a definitive answer to existing controversies. For this reason, the review aims to highlight areas of congruence in the evidence, identify areas where questions remain, and discuss how certain aspects of translational research can be expected to facilitate the transition of this conceptual axis into clinical practice in measurable terms. In this context, the intention is therefore not to achieve finality, but rather to establish a degree of orientation, providing a useful framework through which it may be possible to bridge the gap between mechanistic neuroscience and the emerging neurovascular precision medicine.

## 2. Anatomical and Structural Foundations of the Glymphatic—Venous Axis

The glymphatic–venous axis is a complex anatomical system that integrates peri-arterial inflow of CSF with interstitial exchange, perivenous outflow, meningeal lymphatic drainage, and perineural outflow to establish a connectivity from the brain to systemic circulation and immune surveillance. It is not a conduit system per se, but an integrated hydrodynamic and cellular system whose effectiveness relies on the excitability of vascular pulsatility, astroglial polarity, extracellular matrix porosity, venous compliance, and lymphatic patency [25]. The recent revelation since 2022 provides not only the structural diversity of these trajectories, but also vulnerability and susceptibility due to aging, disease, and surgical manipulation [26]. This section is an anatomical endeavor, integrating developmental origins, regional specialization, biomechanical forces, clinical correlates, and conceptual challenges to characterize the glymphatic-venous axis both as a functional prerequisite and as a pathological weakness.

### 2.1. Periarterial Influx Pathways: Developmental Gateways of Entry

Periarterial spaces, recently termed by Virchow and Robin, are now accepted as the primary entry points of CSF into the parenchyma. In anatomical terms, they occupy the space between the vascular adventitia and the glial limitans externa, with a cross-sectional width depending on the caliber of the vessel. In large penetrating arteries, those are sometimes large enough to allow for sleeves averaging 40–100 μm in large humans, while distal arterioles are sparse sheets narrower than 20 μm, leading to convective inflow limitations. From a developmental standpoint, peri-arterial corridors were established postnatally in tandem with vascular branching and astroglial ensheathment [27]. In the neonatal period, the incomplete establishment of perivascular sleeves likely contributes to the greater incidence of hydrocephalus and white matter injury, as clearance routes have not yet fully matured. Comparative evolution emphasizes that interspecies differences exist: relative to the size of the vessel, rodents have a much larger periarterial space, which likely contributes to the more prominent glymphatic signature in experimental models compared to clinical imaging [28].

Biomechanically, periarterial inflow should be modeled in a continuum of oscillation, where high frequency oscillation is cardiac pulsations and CFD and finite element models indicate slow oscillation vasomotor pumps (90% of the cerebral blood vessels, yielding a glial–vascular interface mediating the glymphatic transport. AQP4 channels are located near the astrocytic endfoot membranes and bound to the basal lamina, facilitating rapid, bi-directional flow of water [29]. The anatomical mechanism that facilitates effective clearance is the polarity of the AQP4 channels; AQP4 channels are found in clusters at perivascular membranes on the side where the periarterial space is located [30].

The polarity mechanism is a dystrophin-associated protein complex that anchors AQP4 to the end-foot basal lamina. If the dystrophin complex is not present, AQP4 pools horizontally across the astrocytic cell body, and transcellular adhesion subsequently inhibits directional water transport mechanisms. (2024) Immunofluorescent resolutions indicate that the most polarized cells exist in hippocampal astrocytes, followed by cortical areas and diversity within thalamic areas. Hippocampal networks potentially sensitive to proteinopathy in Alzheimer’s disease are also subject to regional differences in polarization [31].

### 2.2. Astrocytic Endfeet and Aquaporin-4 Polarization: The Molecular Switchboard

Transcriptomic studies identify diurnal oscillations of AQP4 mRNA and endfoot clustering, with the strongest polarization and clearance capacity occurring during sleep. The ongoing noradrenergic tone suppresses AQP4-mediated flux during wakefulness, and AQP4 reestablishes during sleep or sedation. These data connect clearance anatomy to chronobiology, and effectively situate the sleep behavior of a civilized society as structural initiators of clearance [32].

Sex and hormonal differences provide additional avenues for the regulation of astroglial polarity. Treatment with exogenous estrogen increases AQP4 endfoot clustering and vascular compliance, and exogenous testosterone inhibits both. Clinical imaging provides sex-dependent differences in perivascular space volumetric loads so that post-menopausal women are on an accelerated path of clearance dysfunction, potentially from a shutdown of estrogen. It is likely that hormonal variables may modulate the structural properties of the anatomical clearance tolerance [33].

### 2.3. Interstitial Pathways and the Extracellular Matrix: A Dynamic Microenvironment

The interstitial space is basically the space where the CSF floods the matrix post AQP4-ectopy. This matrix comprises hyaluronic acid, proteoglycans, glycosaminoglycans, and peri-neuronal nets to design the tortuosity and porosity of the space. Particle diffusion is more than merely entering and exiting this space, as solute < 1 kDa diffuse freely, and larger proteins would take advantage of some convection of intravascular fluid based on arteriolar-to-venous gradients [34].

The ECM is a dynamic component. During neuro-inflammation, reactive astrocytes secrete proteoglycans that hypertrophy the ECM and prevent convection. Ischemic stroke accelerates matrix degradation and solute flow, but it also creates a glial scar that obstructs clearance. Trauma-induced reorganization of the ECM can produce ionic displacement of potassium and sodium, which influences clearance. Increased deposition of perineuronal nets in the hippocampus and frontal cortex may also arise from hyper-tight exits, as the proteins are not removed during degenerative activity, providing a plausible mechanism for more preferential amyloid and tau deposition in these areas [35].

They propose that interstitial convection alone has a clearance rate of 0.05–0.1 μL/min per gram of tissue, which is several times faster than diffusion. These results suggest that ECM is not only a scaffold structure, but also an important regulator of clearance kinetics. Initial therapeutic uses of ECM modification, including enzymatic degradation utilizing hyaluronidase or localized metalloproteinase activation, showed some benefit in stimulating clearance in preclinical studies [36].

### 2.4. Perivenous Efflux: Collapsible Exit Corridors

Again, the preferential perivenous location of efflux is due to the cross-sectional area being much smaller than that of periarterial routes, and structurally, it is the most compliant. As a metric, perivenous sleeves can range from 15 to 30 μm in the human brain and can be virtually eliminated with increasing venous pressure. Efflux routes merge to the dural venous sinuses, which have meningeal lymphatic vessels lining them [37].

Pathophysiologic failure of perivenous drainage is quite general in this context. For example, chronic venous pressure in IIH patients is generated by transverse sinus stenosis, and they develop venous channels (that would have drained peritisue fluid and intrathoracic pressure) that collapse, leading to upstream venous pressure resistance. In venous thrombosis, efflux is entirely abrogated, and this is visible through imaging with regard to solute retention. A mild increase in venous rigidity in normal pressure hydrocephalus would lead to an impediment, or cessation of, clearance. Clinical events have highlighted the translational population experience; venous sinus stenting not only normalized intracranial pressure but restored glymphatic tracer clearance, linking the anatomy of the procedure to the physiology of clearance [38].

### 2.5. Meningeal Lymphatic Vessels: Immune Bridges of the Axis

MLV was rediscovered in 2015 and continues to be experimentally evaluated. Currently, MLVs are anatomically part of the clearance system, adjacent to dural venous sinuses and dural arteries, and will drain into deep cervical lymph nodes, which serve as both routes of solute drainage and routes of antigen drainage. MLV are 30—to 150 μm, and those velocities are micrometers per second [24].

Functionally, MLVs are the last common pathway of efflux. Ablation studies in 2024, the targeted surgical epithelialization of MLV accelerated amyloid and ultimately caused cognitive impairment in a mouse model of disease. In humans, we have seen candidacy MLV along the superior sagittal sinus and transverse sinus, and even view the tracer with regard to non-contrast [39].

MLV are clinically remodeled in various entropic environments. They become smaller and therefore less proficient at flow as we age; fibrotic as we have antral disease; and we accept that gliomas secrete MLV to utilize efflux drainage as they migrate to cervical lymph nodes. Autoimmune disease utilizes antigen drainage through the MLV form, or consider multiple sclerosis, where the immune system is not deficient but rather clearing. Neurosurgical procedures have extremely damaging implications; dural resection and venous sinus reconstruction may damage MLV, affecting all observed draining impacts and, possibly, immune monitoring.

### 2.6. Perineural Routes: Evolutionary Safeguards and Fragile Exits

In addition to the venous and lymphatic pathways, perineural pathways provide an alternative form of redundancy. The olfactory pathway, which passes through the cribriform plate, has been studied the most; optic, trigeminal, and spinal perineural pathways also help. In an evolutionary sense, these types of redundant exits may facilitate clearance when central pathways are blocked [12].

Clinically, perineural pathways are some of the least robust. Viral infections, including SARS-CoV-2, disrupt olfactory outflow and are associated with anosmia and cognitive decline. Skull base injury interrupts olfactory clearance and places one at risk of both neuroinflammation and meningitis. Experimental occlusion of olfactory pathways promotes accelerated amyloid deposition, indicating that the failure of perineural clearance is a contributing factor to prodromal neurodegeneration. Anosmia is one of the earliest symptoms noted in Alzheimer’s and Parkinson’s disease, and likely mainly represents failure of clearance, not just cell death [40].

### 2.7. Systems Integration, Regional Vulnerability, and Quantitative Anatomy

The glymphatic–venous axis is viewed as a continuum; therefore, a failure in one location will propagate throughout the system. Computational modeling in 2024 shows that a decrease of 2 mmHg in venous sinus pressure leads to a 50% reduction in periarterial inflow and a one-third reduction in interstitial clearance. In contrast, a 10% increase in vasomotor oscillations has a capacity to increase glymphatic inflow by 40%—these quantitative assessments show the sensitivity of clearance anatomy to pressure [41].

Regional vulnerability corresponds to structures. The hippocampus’s dense AQP4 polarization and limited efflux routes predispose amyloid accumulation. Posterior fossa dependence on transverse sinus outflow is impaired in IIH. The olfactory system depends on perineural pathways that are compromised by viral infection or injury. Periventricular white matter, where the density of ECM coincides with venous drainage, is vulnerable in small vessel disease. These patterns map directly onto disease topographies, substantiating clearance failure as a common denominator [24].

### 2.8. Pharmacological and Oncological Implications

**Figure 1:**
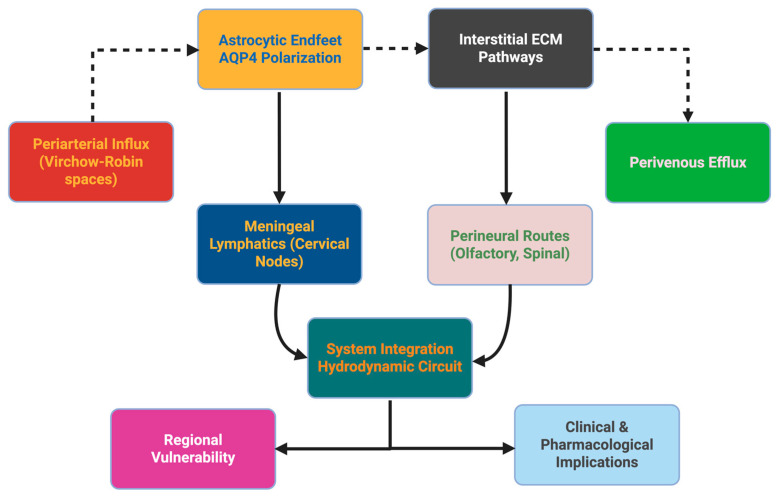
Anatomical and structural foundations of the glymphatic–venous axis. The schematic summarizes the principal clearance pathways, beginning with periarterial influx, astrocytic aquaporin-4 polarization, and interstitial extracellular matrix transport, and converging through perivenous efflux, meningeal lymphatics, and perineural routes. These components integrate into a pressure-sensitive hydrodynamic circuit whose efficiency determines regional vulnerability and influences pharmacological distribution. A dashed side box denotes the major controversies and unresolved questions that remain in the field. The figure is intended as a conceptual framework to complement the anatomical details provided in Section 2.

The integrity of the anatomical clearance axis has implications for physiologic and therapeutic distribution. Intrathecal chemotherapy depends on perivascular/perineural pathways, whereas nanoparticle drug carriers use periarterial convection. Disruption of anatomical clearance in tumors or edema may help to explain heterogeneity in drug delivery. In neuro-oncology, tumors surrounding venous sinuses or astent mLVs change clearance anatomy directly, while glioblastoma induces angiogenesis that forms pathological perivascular spaces for clearance. Metastatic tumor cells utilize mLVs as highways for spread into cervical drainage, providing a reframing of cancer biology through clearance anatomy [42]. To consolidate these anatomical concepts, Figure 1 depicts a schematic representation of the glymphatic–venous axis, which describes periarterial influx, astrocytic aquaporin-4 polarization, interstitial matrix pathways enabling perivenous efflux, meningeal lymphatic drainage, and perineural pathways, thereby illustrating a pressure-sensitive hydrodynamic system. Downstream implications are noted for regional vulnerability and therapeutic distribution. The side box denotes the major prevalent arguments and unknowns. This integrative schematic is meant to serve as a conceptual framework, not to replace the previously mentioned details and their complexities.

### 2.9. Controversies and Knowledge Gaps

While we have made strides, there are significant gaps in our knowledge. Some have stated that peri-vascular transport is primarily diffusion in humans; thus, those investigators took issue with the extrapolation of rodent models. The function of capillary basement membranes—barrier, or conduit—is currently unknown [43]. Quantitative imaging biomarkers are currently subjective numbers that are unstandardized between sites and limit reproducibility. The degree of active modulation of cerebral lymphatic efflux by venous sinuses is undefined, as is the modulation of the balance between CSF-induced convection and parenchymal diffusion in large human brains. This empirical exploration underscores both the vulnerability and the potential of the field, but it is hopeful that the glymphatic-venous axis will soon take the place of neurovascular science [44].

## 3. Molecular and Cellular Regulators of the Glymphatic–Venous Axis

The clearance of solutes via the glymphatic–venous system is due not only to the anatomical structure but also to the actions of molecular conduits, rhythmic vascular driving forces, mural contractile cells, immune-endothelial interactions, lymphatic pump action, and venous boundary conditions. Collectively, much has been learned about these regulators recently; however, much remains circumstantial, and the translational applicability to human physiology is questionable. We cover current evidence, noting the level of mechanistic detail, biological variability, and clinical significance of each.

### 3.1. Astroglial Water Channels: Polarity, Isoforms, and Biochemical Regulation

AQP4 remains the focus of perivascular water movement, but new evidence indicates that its function is almost entirely a function of localization. In non-injured tissue, the AQP4 is polarized to astrocytic endfeet, anchored by dystrophin-associated multi-protein complexes (a-syntrophin, dystrobrevin), an important element of the intracellular network. When polarities of the cellular networks are disorganized, such as after a traumatic brain injury, a-synucleinopathy, and aging, the half-life of clearance of a tracer is increased, with a cause-and-effect shown, but a mechanistic connection is not yet shown [45].

The types of isoforms add an additional layer of complexity. The M23 isoform produces orthogonal arrays of particles (OAPs), structured aggregates characterized by high water permeability. While the M1 isoform disaggregates the structure. Ischemia and inflammation may result in a shift from the M23 isoforms to the M1 isoforms, which may further decrease convective transport. Regulatory mechanisms via post-translational modifications add additional levels of regulation [46]. Phosphorylation and ubiquitination of AQP4 can alter the route and trafficking of AQP4, and some work in 2024 may show that the reappearance of polarized AQP4 following a cell stress is dependent on its phosphorylation status. Elements of the cytoskeleton, such as actin and spectrin, also stabilize AQP4 domains and connect mechanical stress to endfoot stability [47].

Clinical situations can be viewed as “natural experiments”: The presence of AQP4 auto-antibodies in area AQP4 leads to the triggering of circulating AQP4 specificity, resulting in the molecular displacement of endfoot localizations and function, thereby contributing to an acquired model of glymphatic failure. Collectively, this study indicates that AQP4 regulation is multifactorial, which can include scaffolding proteins, isoform ratios, metabolic state, and post-translational modifications, any of which might lend themselves as migraine targets.

### 3.2. Rhythmic Drivers of Clearance

Non-Glymphatic perivascular flow is quite a bit modulated by various frequencies of oscillatory drives. In one study (2025), norepinephrine waves during non-REM sleep were temporally coupled with slow vascular activity, and the output of blood volume changes in states of low cerebral blood flow. These events were also associated with increased CSF flow; blocking norepinephrine waves through pharmacologic sedation reduced clearance while the subject was unconscious, suggesting that the physiological processes of sleep cannot be streamed or replicated with sedatives [7].

Cardiac pulsatility provides diastolic arterial motions; cardiac movement in arteries diminishes with vascular diastolic pressure, as in cases of cardiovascular restoration, aging, and hypertension. Respiration provides the noisy signal, in arterial motion experiments all trial on experiments actually show that masal respiration is superior for clearance related experiments, thus why we suspect to ventilation mechanisms, and different qualities are related out of the primary; respiration is significant to clearance System, ICU in critical care; Non-Glymphatic perivascular flow is perhaps modulated autonomically, as sympathetic dominance likely reduces the amplitude, and tone of vasomotion, and parasympathetic activation has the opposite effect [48].

In addition to slow rhythms, perhaps rapid oscillatory activity is relevant too? Mouse modeling suggests that sensory entrainment could enhance amyloid clearance at gamma frequency to 40 Hz, perhaps by synchronizing hyper-excited neuronal clusters with cerebral vascular compliance. The exact purpose of REM sleep is still a mystery, and there are some who suggest that clearance is impaired in this state or NREM, or that it might have a regional specific contribution. There is a complexity to considering state-dependent clearance regulation [49].

### 3.3. Pericytes and Microvascular Gating

Pericytes are a newly apparent unit that mediates clearance at the microvasculature. Pericyte-mediated calcium oscillations and vasomotion have been demonstrated using imaging and electrophysiology. Pericytes Ca^2+^ oscillations appear to be due to L-channel calcium channels and TRPC3 and drive capillary vasomotion. Small shifts in pericyte calcium dynamics can be an important contributor to the local resistance and thereby variability of clearance to cortical areas near the site of enhancement [50]. Perivascular fibroblasts may also change flow by secreting extracellular matrix, which increases the stiffness of the perivascular sleeve. These studies are descriptive, preliminary, but suggest a possible mechanism of clearance regulation, expression of large vascular vasomotion, and microvascular clearing through gating that modifies your regional volume flow [51].

### 3.4. Immune and Endothelial Interactions

There are further possibilities for perivascular macrophages to play a more active role as solute conduits, as evidenced by similar experiments where the depletion of macrophages lowered tracer and elevated stimulation levels. Microglia, to make it more complicated, are likely quite far from perivascular sleeves and could be playing a somewhat distant role using cytokines, complement activation, and synaptic pruning and signaling via the meninges. Endothelial cells are also able to release VEGF-A and TGF-β to modify permeability through an endothelium, or at least induce perivascular adhesion, but can become dysadaptive with persistent stimulation [52].

In addition, adaptive immunity has been brought to the forefront of developments. B-cells, which have been seen continuing to cluster outside of meningeal lymphatics, also make local antibodies and possibly even have an impact on local draining ability. This brings into play that the glymphatic-venous axis plays a role in addition to clearance, but may be, an interface for immune surveillance with both neuroinflammation and autoimmunity potential [53].

### 3.5. Lymphatic Contractility and Plasticity

The “special” candidates of meningeal and cervical lymphatics, previously identified as passive, are truly rhythmic contractions. The 2024 studies demonstrated rhythmic contraction of lymphatic vessels at approximately 3 Hz, with slower rates and amplitudes measured in older specimens. The addition of chemical agents (prostaglandin-F2α) rhythmically contracted lymphatic vessels, and further, the evidence is mounting that lymphatic vessels can act as pumps. VEGF-C signaling has been delineated as thought to be the primary mediator of lymphatic growth and maintenance [54]. Immunotherapy with recombinant VEGF-C demonstrated reversing a lymphatic depot in studies of neurodegeneration and sepsis. The rate of lymphatic loss with aging correlated with loss of VEGF-C. Additionally, lymphatic endothelial cells may serve as antigen-presenting cells, thus moving them from fluid transport to regulating immunity. Overall, the data imply that lymphatic vasculature is more dynamic and adaptable than we realized, yet linguistically still lies far from translational to humans [55].

Venous hydraulics and the systemic context. Venous outflow is the single boundary condition. Mathematical modeling of transverse sinus stenosis showed that an area stenosis still increased venous pressure and created a turbulent venous flow, both of which can unbind perivenous cuffs. Stenting was shown to restore venous pressure, but we still do not know if clearance is restored [56]. Systemically speaking, obesity, elevated central venous pressure, and ventilatory settings following critical post-op settings may raise venous pressures and impair clearance. The extent to which venous congestion can induce neurological pathology through this pathway is uncertain, but surely it is becoming an increasingly important consideration [57].

### 3.6. Venous Hydraulics and Systemic Context

For some, time may serve as a barrier to the efficiency of treatment with circadian effects. AQP4 expression and polarity peaks at night, in nocturnal animals, coinciding with NREM sleep. Circadian misalignment reduced tracer transit in studies of sleep deprivation and shift work, suggesting that even clearance efficiency might be an intrinsic variable to circadian rhythms [32]. The pharmacological effects vary tremendously. Ketamine/xylazine anesthesia increased brain tracer transit, and zolpidem would decrease brain tracer transit, likely through reducing norepinephrine pulsation. Other medications, especially medications with different mechanisms, presumably have lesser effects. Diuretics reduced venous pressure, and possibly increased effluent; SGLT2 inhibitors may alter physicochemical osmotic gradients, which may alter interstitial fluids; anti-muscarinics, which reduce vasomotion, etc. All of this illustrates the point that clearance pathways have been modified by pharmacological agents, which probably are not the intended pathway of clearance [58].

### 3.7. Quantitative Modeling and Imaging

Modeling has been pursued to create a quantitative estimate of clearance. Modeling theorizes that a 20% increase in arterial tone may decrease the speed of convection by 40%. The half-clearance times of tracer probability are much longer in humans than in rodents, possibly implying that both scaling and anatomy may also be important factors in clearance efficiency. Pressure gradients in rodent models have been estimated to be 1–3 mmHg, but comparable human data are extremely limited [59].

Imaging biomarkers are slowly advancing. Diffusion tensor-based imaging metrics, such as ALPS, are likely sensitive to perivascular transport but highly dependent on technical parameters. High-resolution MRI sequences have been used to visualize meningeal lymphatics in vivo, which could potentially allow for a non-invasive evaluation of lymphatic contractility and remodeling. These techniques are promising but would need validation before general use in clinical trials [60].

### 3.8. Debates and Uncertainties

There are still many fundamental questions unanswered. The extent to which clearance is achieved by convection relative to diffusion in humans is still disputed, with support on both sides. There are problems surrounding the permeability of capillary basement membranes to macromolecules. These are compounded by species differences: rodents demonstrate efficient clearance, but this may not translate to primates or humans, where the volume of white matter and the extent of cortical folding modify perivascular geometry. Mechanisms of human brain clearance are even less understood than animal models, and while there is some evidence that clearance is more efficient in the cortex than in the brainstem, systematic comparisons are lacking. Possible sex differences, potentially influenced by vascular compliance after menopause, have only recently been hinted at [61].

### 3.9. Therapeutic Perspectives

Many of the experimental manipulations may be capable of modulating clearance. These include methods for restoration of astroglial polarity (e.g., GLP-1 receptor agonists), promotion of oscillatory drivers (sleep protection, gamma entrainment, respiratory synchrony), enhancement of lymphatic function (PGF2α, VEGF-C), and alleviation of venous congestion (stenting, optimized ventilation strategies). These methods are presumed to be mechanistically distinct, indicating that possible combinations of methods may be feasible. The majority of evidence remains in the preclinical proof stage; strategies will require judicious translation to humans. Regulators of the glymphatic–venous axis comprise the molecular, rhythmic, immune, lymphatic, and venous dimensions [9,62]. They seem to work in concert, with sensitivity to sleep, circadian rhythms, pharmacology, systemic physiology, and pathology. While recent work has provided unprecedented mechanistic depth, much is still uncertain, especially for human physiology, regional specificity, and translatability. A fuller characterization of these regulators may provide avenues for targeting clearance; however, the area is still in an embryonic stage [63].

## 4. Pathological Failure of the Glymphatic–Venous Axis

The disorder of the glymphatic-venous axis is not a single lesion but a failure of the pan-network of astroglial depolarization, extracellular matrix stiffening, perivascular circumscription, back pressure on venous returns, and decreased contractility of the lymphatics. The failures can be focal or diffuse, bidirectionally integrated into astroglial, vascular, and immune circuits. A convergent hypothesis has emerged that defects in clearance may be a possible underlying basis for neurologic, psychiatric, and systemic disease.

### 4.1. Neurodegenerative-Protein-Aggregation Syndromes

Of the neurodegenerative diseases, the clearest situation is that of Alzheimer’s disease: its specificity of defect in clearance predicts the toxic storage of proteins. Single cellular and spatial osmological studies reveal the loss of dystrophin complex anchoring and also AQP4 depolarization of perivascular astrocytes in the proximity of amyloid plaques; both are relevant in plaque burden and cognition [64]. The stiffening of perivascular matrices from glycation or increased storage of lactate increases hydrodynamic resistance, while ultra-high field tracer imaging points to a regional factor in clearance half-time [65]. Sleep-dependent oscillations in norepinephrine and gamma-40 Hz entrainment lead to restoration of vasomotor control and enhanced amyloid clearance [66]. All these data are suggestive of a possible physiological intervention. Similar facts are found in the situation of Parkinson’s, where defective clearance of α-synuclein associated with defective perivascular pulsatility (especially during the prodromata of REM-behavioral disease) activity predicts eventual motor onset [67,68].

Defective glymphatic transport is also a factor in tauopathies and prion diseases, where obstruction to the perivenous clearance leads to rapid stereotyped instaurations of the propagation that perivascular drainage layout can dictate stereotyped proteinopathy propagation [69]. All these data stress the possibility of clearance malfunction as a potentially remediable causal factor of the proteinopathies rather than a secondary by-product

### 4.2. Errors in Clearance Related to Vascular and Trauma

Ischaemic and hemorrhagic strokes acutely cause collapse of the perivascular sleeves, from pericyte loss, changes in astrocyte isoforms (M1 preponderance of AQP 4), and deposits of hydrocarbon compounds related to hemoglobin affecting outflow [70]. The chronicity of small vessel disease affects changes to fibrotic basement membranes and thus compliance and in different degrees of venous stenosis, development of abnormality affects backpressure and delivery of local clearance [71]. Head injury produces immediate AQP4 mis-sorting and a permanent decline in transport performance relates to cumulative loading of impact [72]. Perivascular distribution of tau and AQP4 breakdown in chronic traumatic encephalopathy, suggesting misaligns possible that stress can provoke clearance loss over time [73].

### 4.3. Neuroinflammatory, Infective, and Neoplastic States

The inflammatory demyelinating illnesses, e.g., multiple sclerosis, display a pathophysiology-related state-induced slowing of tracer passage clear during relapse by demonstrating lymphatic constriction, as noted also by changes in venous compliance speech [74]. Autoimmune encephalitides are associated with IL-6 and AQP4 disorganization. Infective agents such as tuberculous meningitis, neurosyphilis, and SARS-CoV-2 may obstruct lymphatics, affecting the basal vessels or inflamed endothelial possible cause of fortity continuative post-infective cognitive dysfunction [75,76]. Glioblastoma produces an effect on clearance due to hyaluronan-rich matrix stiffening, loss of AQP4, and uptake of AQP9 in the affected region, peritumoral astrocytes, and ENG-mediated effects on lymphatic impairing processes through local edema. Surgery, radiotherapy, and scarring modify venous and lymphatic field geometries, further indicating the necessity of preservation of drainage corridors in space in oncological and neurosurgical medicine [77]. Systemic comorbidity, hypertension, diabetes, hepatic or renal insufficiency, primary effect endothelial thickening and glycocalyx stiffening, may cause blunted convective power. Agents which produce improvement in stiffness of endothelial responses or reduce venous load (e.g., SGLT2 inhibitors and venous sinus stenting). Also exhibit similar improvement in clearance indices in experimental models used [78,79,80,81].

Psychiatric and sleep states similarly damage clearance physiology. Depression and schizophrenia show an increase in the size of perivascular spaces with enhancement of lactate signals, while desolations are possible due to lack of slow wave missleep, or circadian types of illness engender diminished glymphatic passage [82]. These links between metabolic or behavioral states induce clarification of mechanism types which dictate the state of the dynamics of fluid in the brain.

### 4.4. Integrative Mechanisms and Translational Perspectives

From among the various modes, four mechanisms chiefly emerge: the astroglial loss of complete polarity, the enhanced rigidity of the extracellular matrix, the hypocontractile state of the lymphatic effects, and the resistance at the venous outlet. State of each of types of mechanisms connectable to experimental and reversible ways by bringing about restoration to the state of sleep, by bringing about the synthesis of lymphangiogenesis by use of VEGF-C, prostaglandin physiology, and mechanical decompression of the venous effluent [83,84,85]. Recognition of such pathways improves the precision phenotyping of clearance endotypes, since they may lead to the same therapeutic endeavors, all of which are applicable to broad classes of neuronal and systemic illnesses.

The contrast of these various types actually coalesce into a unified, mechanistic movement of understanding and possibility of translation. The reconstruction of homeostasis in the matter of fluid passage will enhance cognition possible cognitive resilience but will in fact decrease continuous production of disease and load of disease processes in the complete range of neurodegenerative, vascular, and psychiatric states of illness [86,87].

Table 1 intends to show diagrammatically and profusely the chief and principal methods of approach to the methods and techniques of method, emerging advanced and new MR metrics, molecular panels, and improved wearables, which will give the possibilities of the translation of state-of-clearance physiology to assessable clinical endpoint measures.

## 5. Diagnostic and Biomarker Frontiers

Accelerated advancements in the transition from conceptualizing the glymphatic–venous axis to the use development of physiological diagnostic technologies have occurred due to advances in imaging, bi-fluid science, electrophysiology, and computational modeling. These areas of progress have now made clearance a topic of mechanistic neuroscience and a candidate clinical biomarker across neurology, psychiatry, neurosurgery, and intensive care. By connecting normative databases, novel modalities, translational anchors, and a global equity approach, this section of the manuscript presents the multiple dimensions of clearance diagnostics and prospects of standardizing applications in the future.

### 5.1. Magnetic Resonance Imaging Biomarkers

#### 5.1.1. Diffusion Metrics

Diffusion MRI provides robust, non-invasive biomarkers. For adults, the ALPS index has normative values of 1.4 through to 1.6, with inter-center variation of 10–12% and a decline of under 0.005 units per year. For pediatrics, these values are higher (i.e., 1.8 to 2.0), consistent with the peak clearance measures associated with developing synaptogenesis. For comparative data, the ALPS for rodents are about 20% higher than humans, consistent with their faster metabolic cycle, while initial primate data is between the two [94]. For children, sex differences between the ages of 2 and 12 are significant until menopause, post which clearance improves. Ethnicity differences of about 8 to 12% have been noted, though studies are still updating them. Beyond this, 7T diffusion spectrum imaging is showcasing deep perivascular anisotropy with structures below 0.6 mm, and the microstructure anisotropy is mapped to clearance pathways [95].

#### 5.1.2. Tracer Kinetics

Tracer-based MRI allows for dynamic second-order measures. For rodents, clearance half-time is 2–3 h (i.e., youth) and 6–8 h (i.e., aging). For humans, ultra-low-dose gadolinium and intranasal nanoparticles at safe mapping have an influx of 30 to 60 min and clearance slopes of 0.03 to 0.05/min. Medial temporal lobe prognostication of regression threshold retention > 0.2 will predict cognitive decline, and the ability to replicate to shorter time intervals is forthcoming. Non-contrast spin labeling and isotropic phase contrast MRI are evolving toward more widely utilized, safer imaging [96].

#### 5.1.3. Physiological Gating

Clearance is limited by pulsation forces; ultrafast MRI, temporally congruent to one’s cardiac and respiratory rhythm, yields a synchrony coefficient in health from 0.7 to 0.9 to 70% predictive accuracy). Intraoperative indocyanine green shows contractile function, and the next wave of photonic flow probes can permit micrometer velocities to be measured during surgery; this will facilitate mechanistic validation of meningeal clearance [97].

##### Development, Sex, and Population Variability

Clearance progresses across the life-course from neonatal immaturity, postnatal development, peak development in early childhood, plateau in early adulthood, and a decay associated with aging. Sex-based differences emerge at puberty, which is a beneficial estrogen phase until menopause. Hormonal manipulation studies demonstrate that estrogen administration promotes vascular compliance and AQP4 anchoring, while androgen suppression interrupts physiologic pulsatility [98]. Ethnic and environmental inter-individual differences, including altitude physiology, may introduce a clearance ‘fingerprint’ profile. Pediatric conditions such as hydrocephalus or seizures have shown clearance ‘fingerprints’ that predict treatment efficacy. Composite indices, AI-enabled profiling Integrated analysis across modalities. Composite algorithms of MRI half–times, EEG slow-wave density, and plasma metabolites accurately detect early cognitive decline with >85% accuracy. Machine learning is also now able to capture non-linear relationships between pulsatility and contractility, as well as systemic determinants (HbA1c and arterial stiffening). Digital twin modeling can estimate clearance under patient vascular geometries to anticipate therapeutic effects. The clearer vision of a “passport” to clearance and updating at every moment of life, with early assessments of vascular risk, would be the premise for neuro-prevention precision [99].

### 5.2. Clinical and Systemic Anchors

Diagnostic clearance is tied to the multi-sector clinical work represented by clearance indices. In the ICU, for example, positive end-expiratory pressure and endotracheal intubation will reduce clearance indices, whereas prone positioning will improve them. In neurosurgery, our ability to monitor intraoperatively provides us with possible predictions of post-surgical swelling or intracranial pressure. In oncology, clearance PET can measure peritumoral edema, while on-target immunotherapy is ongoing. In psychiatry research, EEG-clearance composites have been investigated in insomnia, depression, as well as early pilot studies in bipolar disorder, and PTSD [100]. Finally, since clearance is a systemic physiology, we would expect arterial stiffness, glycemic status, uric acid status, or renal status to impact efflux longitudinally [101].

### 5.3. Pharmaceutical and Translational Trial Relevance

Clearance metrics have also been used for the first time as putative surrogates for endpoints in drug development. An example in brain Canada anti-amyloid and anti-tau antibody trials clearance metrics would have been endpoints in study reports (value and risk of ongoing tissue edema or anti-therapeutic effects). Synthesis of investigational agents with some neuroprotective potential is interested in clearance-indices treatment effects through products like ALPS (astrocyte and neural progenitor stem cells) and exosome-derived biofluid and clearance b xi. A sound focused ultra-sound approach (blood–brain barrier) to assess CNS drug permeability may yield imaging clearance to support treatment effects. Therefore, there will be growing interest from regulators about clearance as a translational credential. Standardization, ethics, and regulatory perspective [102].

Consortia like CLEAR-MRI and CHAMONIX are creating standards for acquisition and assembling global datasets. Regulators are starting to accept clearance indices as exploratory trial outcomes, though much harmonization work is needed [103]. Ethical considerations give pause for intrathecal gadolinium, a launching off point for intranasal nanoparticles and print modalities without contrast. Population-based screening will rest on platforms that are safe and cheap, like ultrasound, EEG, and biofluid assays, which align with just and equitable population-level clearance monitoring [104].

### 5.4. Conceptual Integration

Diagnostic schemes could be constructed on structural (diffusion anisotropy, lymphatic patency), dynamic (tracer kinetics, pulsatility synchrony), and molecular (proteomics, metabolomics, extracellular vesicles) tiers. The diagnostic tiers each have distinct strata of clearance physiology and are not intended to be mutually exclusive or competing, but complementary. Synthesis across strata, across the lifespan, and across populations could lead to clearance being part of every neurologist’s standard of care in neurology, much like perfusion and electrophysiology are today. There is as much need in the emerging field of open-data repository as well as AI-based harmonization pipelines to assure reproducibility from the center or vendor [105].

### 5.5. Global Accessibility and Equity Considerations

The promise of clearance diagnostics needs to be universally accessible. Advanced MRI and PET may forever be the realm of high-resource centers to facilitate scientific discovery, but portable EEG–NIRS hybrids, Doppler ultrasound, and plasma assays provide local and distributed elements in lower resource settings. Populations at high altitude even provide natural experiments of variability of clearance, as well as highlight the need for normative studies on a variety of physiologic traits. Equity-derived translation may also provide an assurance that clearance is generalized horizontally versus vertically, and not just limited to a subgroup or biomarkers of brain health [106].

Progression from high-field MRI to bedside ultrasound, chemical assays to AI-derived digital twins, continues the evolution of the clearance diagnostics. Each of the clearance diagnostic systems offers insights into one tier of physiology; however, only when working interdependently do they render a better picture of physiology, encompassing structural, dynamic, and molecular tiers. Horizontal and vertical levels of harmonization, temporality, and equity-based parameterization of clearance could someday be thought of as analogous to perfusion or metabolism as a core dimension of brain health and disease classification, with implications for neurology, psychiatry, neurosurgery, and beyond [107]. Along with diagnostic approaches, evolving interventions are all the more focused on clearance dynamics. These interventions range from pharmacologic additions, device-based neuromodulation, vascular interventions, and lifestyle-based interventions [108]. To give a homogenized viewpoint on this developing therapeutic area of clearance dynamics approaches, we have attempted to lay out the most expressive approaches in 2024–2025 (Table 2), with their mechanisms of action, timing of action, predicted biomarker effects, and stage of translation.

## 6. Therapeutic Strategies Targeting Brain Clearance Coupling

The glymphatic–venous axis is rapidly transforming from an experimental notion into a translatable enterprise in the therapeutic modulation. A defining feature of this area is its reversibility: clearance dysfunction, unlike most degenerative processes, can be modified at different time periods (minutes, with posture changes; years, through vascular remodeling). Therapeutic interventions encompass the full range from optimizing lifestyle choices to neurosurgery bioengineering to regenerative medicine therapies and trial-ready biomarkers. Each therapeutic approach benefits from interdisciplinarity, as distinct fields (neurology, cardiology, nephrology, sleep medicine, immunology, bioengineering), our respective fields can all coalesce around clearance as an important determinant of cerebral health.

### 6.1. Physiological and Lifestyle Modulation

Sleep architecture is the most powerful innate way of maximizing clearance. Two- to three-fold greater glymphatic flux and ~60% greater interstitial space during sleep when compared to the waking state. In functional brain imaging studies of humans, slow oscillation density (>0.8 Hz) was shown to linearly correlate with clearance efficiency, such that a 10% decrease in the density of slow oscillations was associated with a 12–15% decrease in ALPS indices [118]. Closed-loop auditory stimulation corresponding to the up-phases of slow oscillations produced increases in clearance for the night; transcranial alternating current stimulation (0.75 Hz) produced comparable effects on clearance. Sleep-disordered breathing demonstrates how vulnerable clearance is to physiological disturbance (i.e., obstructive sleep apnea demonstrated ~40% reduction in pulsatility synchrony) [119]. Yet, CPAP for eight weeks restored ~60% of lost clearance capacity; furthermore, cognitive gains were evident. Body posture has additional modulating effects on clearance. Lateral decubitus position leads to 15–20% increased tracer exit velocities versus supine position in rodent and human studies, with longer supine duration leading to 30–40% longer tracer half-times [120]. Circadian alignment also matters with clearance: some rodent studies show nocturnal tracer infusions lead to ~40% increased clearance velocities, and circadian dis-alignment (like a shift worker) shows ~12–15% decreased clearance indices. Therefore, it cannot be argued based on this data that posture and circadian regularity are described as a treatment lever versus a lifestyle factor. Systemic vascular health is expected to influence that clearance [121]. Hypertension was shown to yield about—40% longer tracer half-times, while arterial stiffness (>10 m/s pulse wave velocity) is significantly predictive of expected clearance decreases. Calcium channel blockers promote clearance independent of BP. Notably, calcium channel blockers inhibited perivascular pulsatility. Exercise has a dose-response effect, with repeated studies showing that ≥150 minutes of maximum has been associated with a ~0.1 change in unit ALPS indices [122]. In addition, the slope of each runner’s VO2 max was improved among participants with clearance. Nutritional- just like, high polyphenols and omega-3 polyunsaturated fatty acids diets correlate with decreased astroglial activity, and if improved perivascular compliance of rats after stroke injury without controlled trials clearly [123].

### 6.2. Pharmacological Strategies

For example, AQP4 polarity could be a possible treatment target. In aged rats, the peptides that block lateral polarity switching produce greater perivascular targeting and ~35% greater clearance velocity. Viral delivery of dystrophin anchoring has been a successful target for an AQP4 polarity restoration in some in vivo studies. Nevertheless, appears to have low rates of water dysregulation risk [124].

Avascular pulsatility facilitators could be a potential therapy. Nitric oxide donors can rescale pulse variation amplitude, which achieves ~20% lower tracer escape half-times. Other medications like cilostazol improve perivascular pulsatility, lower amyloidal deposition in the brain, and cognition following preclinical animal studies. Cilostazol is now in clinical trials to prove the efficacy of vascular dementia and clearance measures. Phosphodiesterase-5 (PDE5) inhibitors may theoretically increase venous capacitance, but clinical studies have only recently started. Inflammatory and metabolic approaches provide another resource for pharmacological therapy [125]. Minocycline reduces astrocytic reactivity and improves glymphatic clearance in about 25% animal models of neuroinflammation. Statin therapy reduced perivascular fibrosis. SGLT2 inhibitors restored glymphatic flux in diabetic mice. Metformin improved clearance by about 20–25% through AMPK-dependent vascular remodeling. These physiologic therapies appear to converge in the overlap between neurology, cardiology, and endocrinology [126].

Repurposing agents used in the clinic is also promising. Sleep medications can improve clearance by enhancing slow-wave sleep and providing an indirect physiologic effect. Anti-epileptics that modulate astrocytic gap junctions improve the tandem coupling of the glymphatic flow. Nanoparticle-encapsulated drugs that utilize perivascular inflow increased CNS penetration by up to 40% premised on the glymphatic drainage pathway being not only an outflow system, but a delivery system [127].

### 6.3. Mechanical and Electrical Neuromodulation Approaches

Non-invasive brain stimulation (NIBS) can tap into neurovascular oscillations. Closed-loop tACS can be tied to EEG-detected slow waves and improve perivascular inflow by ~25% in rodent studies, and improve clearance biomarkers in humans. TMS protocols that parametrically match slow-wave frequency appear to improve clearance and are currently being investigated in an early-stage study with tracers. Ultrasound is a mechanobiological therapy. For example, low-intensity focused ultrasound (LIFU) improved the perivascular pulsatility by 20–30% in rat studies [128]. Microbubble-assisted ultrasound transiently opened the blood–brain barrier and improved drug delivery and clearance. Clinical glioblastoma protocols in humans are using this strategy and are analyzing the clearance biomarker criteria as a component of oncological outcomes [129].

Wearable biofeedback clearance systems are a scalable solution within this paradigm. An EEG device, posture sensor, and heart-rate monitors will identify behavior patterns that adversely affect clearance and provide feedback for correcting such behaviors, and promote slow breathing and repositioning. Though in the early stage, these strategies and methodologies could lead to a democratization of clearance therapy, from the specialist centers, to primary care and preventative medicine [130]. Neurosurgical and Interventional strategies. The strongest outcomes for full clearance modulation are neurosurgical. Hydrocephalus with CSF shunting provides a means of clearance reform, and with that, increased pre-operative tracer retention can predict post-operative treatment response at ~70–80% accuracy. The treatment of venous sinus stenosis was developed first for the purpose of decreasing venous pressure [131]. They established an effective treatment that could render about ~30% improvement in baseline efflux and improvements in headache and visual outcomes. Biologics and experimental augmentation to meningeal lymphatics could complement surgical techniques. Various studies demonstrated that delivery of VEGF-C evokes lymphatics and improves clearance, in small animal (rodent) models, and a bovine collagen scaffold seeded with ECs led to superior efflux in a large animal model. These bio-engineering techniques promote implementation in neurosurgery or one day lead to regenerative remodeling of clearance structures. Regenerative and experimental methods are the most unfounded, and therefore, creative [132]. Regeneration via CRISPR-based flip editing was one in vitro study that corrected polarity-based mutations in an astrocytic scaffold, restored AQP4 positioning. Viral vector mediated delivery of proteins which sustain polarity also restored clearance in aged mice albeit human delivery is an unknown. Cell based strategies, like endothelial progenitor cell infusion restores venous capacity and clearance in stroke in mouse models, another [133]. Synthetic human hydrogels mimicking/replacing ECM perivascular conduits improved transport at or near the interfaces in animal models, a bio-materials therapeutic approach. Autologous meningeal grafts with lymphatic endothelial cells also have potential in marrying neurosurgery with tissue engineering. Omics-based therapeutics add an additional layer of precision. The identification of astrocytic and endothelial subclusters by single-cell transcriptomic profiling is critical in clearance. There are case reports of RNA-based therapies directed at the specific subclusters, a shift from global modulation to cell-type–specific repair [134].

### 6.4. Clinical Trial Design and Translational Readiness

Clearance outcomes are becoming incorporated into trials more and more. Both lecanemab and donanemab Phase II studies are utilizing clearance biomarkers as exploratory outcomes; instead of predicting imaging abnormalities related to amyloid. In stroke rehabilitation, clearance indices have been ‘fit’ modeling the trajectory of functional recovery. Clearance MRI is being used to follow patients undergoing venous stenting in cases of IIH [135].

Trial design is changing. Adaptive Bayesian designs with clearance readouts allow early detection of signals, and glymphatic–venous physiology can be simulated using a digital twin. By utilizing data to model clearance, trial protocols can be optimized. Clearance index as an ended measure has been sanctioned as an endpoint by jurisdictions like the FDA and European Medical Agency for regulatory approval, but longitudinal verification will be needed across populations [136].

### 6.5. Global and Ethical Considerations

Clearance therapeutics need to be framed in a global equity model. High-resource innovations such as PET tracers, CRISPR gene therapy, and regenerative grafts will likely be reserved for specialized centers, but clearance can be assisted by universally accessible lifestyle interventions, such as sleep hygiene, blood pressure control, CPAP therapy, exercise, and biofluid assays. Portable electroencephalogram–near-infrared spectroscopic devices and Doppler ultrasound can provide a scalable, inexpensive way to monitor clearance and be adaptable to the community setting and the lower-resource context. There are planetary health factors to consider [137]. Climate change will affect sleep, altitude will affect venous pressure, and job schedules will affect clearance. Addressing these systemic factors would be a sizable measure in terms of promoting clearance therapeutics, but they would come within the borderline of public health and environmental medicine. Ethical concerns would be the risks of repeated contrast imaging; the continuum between therapy and enhancement (especially if clearance is pushed beyond physiologic limits), the implications of gene editing in non-lethal conditions; Open access data and a global consortium would still be needed to stop inequities in validating biomarkers and access to any therapy [138].

**Figure 2:**
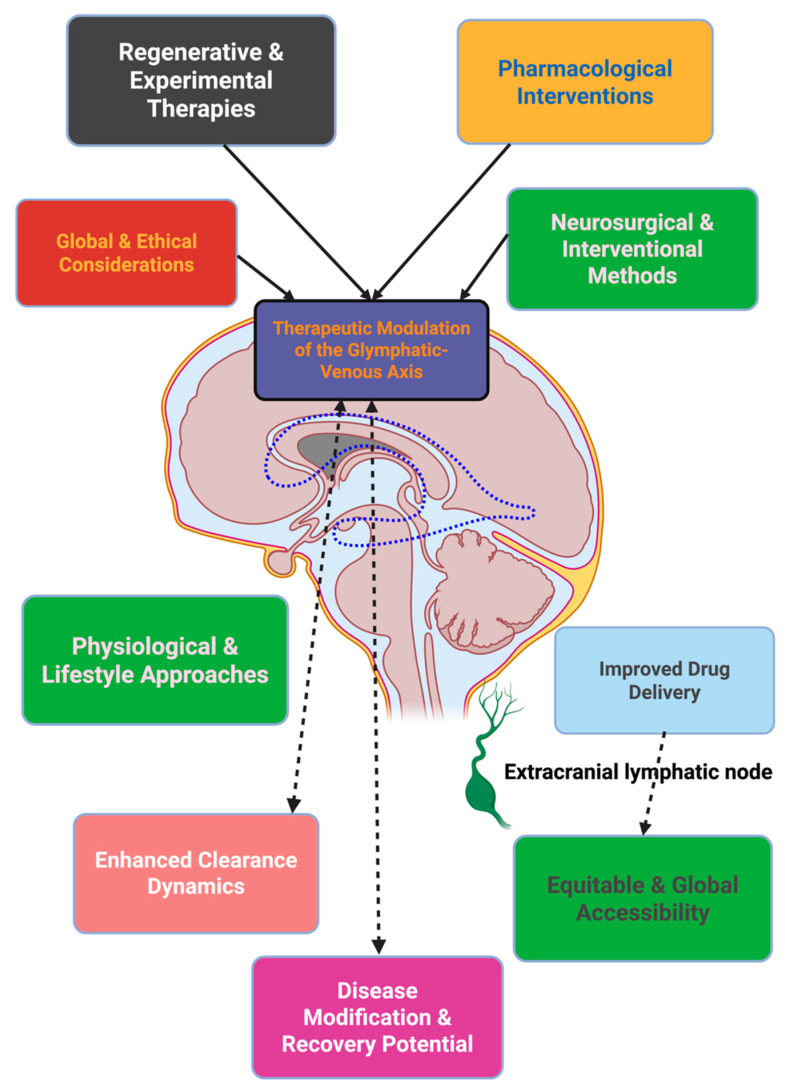
Anatomically grounded schematic of therapeutic modulation within the cerebrovascular–lymphatic clearance network. Interventions across pharmacological, regenerative, surgical, and behavioral domains converge on anatomical pathways coupling periarterial influx, interstitial transport, and perivenous–lymphatic efflux. The coordinated modulation of these routes aims to enhance clearance efficiency, facilitate drug delivery, and promote equitable translational access.

Therapeutics aimed at the glymphatic–venous axis occupy a continuum between posture, circadian regulation, optimization of the cardiovascular system, pharmacology targeting, neuromodulation, surgical intervention, regenerative bioengineering, and molecular repair, the last point distinguishing across a number of timescales, from minutes to years. The real test of whether clearance becomes a mechanistic discovery to the cornerstone of precision neurotherapeutics will be the degree to which clearance biomarkers are used in clinical trials, and clearance-indexed, regenerative, and omics-based therapies; and most importantly, the emphasis on global access to the glymphatic–venous axis [139]. Figure 2 will attempt to depict a graphic representation of the therapeutic modalities harnessing the glymphatic–venous axis, where sleeping, exercising, medications, clinical acts and surgery, and bioengineering and computing converge is in the clearance modulation, with downstream effects on drug-seeking delivery, disease modification, and global therapeutic access.

## 7. Emerging Technologies and Future Directions

The study of the glymphatic-venous axis is very much in, a methodological metamorphosis. Going from an abstract physiological speculation, it is evolving into a domain studied with measurable, modifiable, and increasingly personalized biomarkers. The rapid onslaught of technologies, starting in the 2024s, has built a bevy of technologies—from continuous ambulatory sensors, to AI-assisted digital twins—to study clearance physiology across time, molecular layers, and clinical settings. Within, we highlight the frontier, with a particular focus on the integration into a concerted translational pathway with individual discoveries.

### 7.1. Continuous Human Monitoring of Glymphatic Physiology

One of the most noticed recent innovations is the acquisition of wearable devices capable of monitoring neurofluid dynamics outside of the imaging suite. For example, in 2025, a team reported on wireless scalp-integrated systems that also integrated transcranial impedance spectroscopy, EEG, and hemodynamic sensors. These “headsets” measure parenchymal resistance fluctuations in a manner that accurately corresponds to changes in sleep stages—the relative reductions in resistance during slow-wave sleep, and increases in resistance during arousal [140]. Even more importantly, and subsequently verified with MRI-based contrast clearance benchmarked against “clearance”, the impedance signal showed moderate agreement, identifying it as an achievable ambulatory proxy. In general, the first methods for tracking clearance physiology in humans over sustained periods of weeks to months in a real-world setting, considering circadian rhythm, posture, and environmental exposure as subtle interactions [141].

In time, this will formulate new clinical avenues for research. For instance, the ability of these devices to measure and therefore quantify the effects of melatonin supplementation in shift workers, or the candidate of high-altitude exposure on venous outflow without relying on hospital-based imaging. Furthermore, contrasting biosensor data streams along with a machine-learning classifier has produced predictive scores of clearance efficiency. The transition from episodic imaging to continuous physiology is a significant move to real-time intervention studies in home settings [142].

### 7.2. Sleep Neurochemistry and Vasomotor Coupling

Sleep has long been associated with clearance efficiency, and only recently has it been revealed that there are mechanistic differences. In 2025, work using optogenetics demonstrated that infraslow norepinephrine oscillations from the locus coeruleus modulate vasomotor rhythms, thus driving oscillating cerebrovascular thickness and CSF flux. Inhibition of norepinephrine oscillations led to disrupted clearance, and pacing the norepinephrine oscillations re-established clearance [143]. They provided mechanistic linkages between cellular neurochemistry and systemic hemodynamics, and created a causal pathway from norepinephrine tone → vasomotor tone → glymphatic pulsatile oscillatory clearance. This mechanistic clarity paved the pathway towards a new type of therapy. Closed-loop neuromodulation may promote infraslow oscillations from locus coeruleus norepinephrine during NREM sleep, using focused higher-order electric stimulation, or, via pharmacological entrainment [144]. Moreover, simultaneous EEG-impedance recordings can advance the translation of these therapies to humans. It should be noted, clearance may be directly coupled to these rhythmogram fractions and supports the importance of sleep hygiene, circadian coherence, and targeted pharmacological therapies on neurofluid homeostasis [145].

### 7.3. Rhythm-Based Augmentation of Clearance

The notion that our rhythmic-based stimulation will entrain clearance has now been directly demonstrated in experimental evidence. In mouse models of Alzheimer’s Disease, 40 Hz (gamma) sensory stimulation led to a systemic improvement in memory and reduction in brain amyloid-β, while increasing glymphatic solute flow. Importantly, the reduction in observed clearance was reported to occur within hours to days—substantially earlier than any cognition benefit—thus suggesting that the mechanisms have an engagement pathway vs. downstream change related to disease modification [146].

This lays the foundation for multimodal protocols for rhythmic therapies with gamma entrainment and infraslow oscillatory facilitation. The feasible combinations of slow vascular-driven pumping in the interface with the CNS and gamma-based coupling of neural oscillations suggest that clearance will be responsive across networks of frequency, with each frequency engendering a different, but convergent, pathway. From a clinical standpoint, rhythm-based therapies have obvious advantages of being non-invasive, titratable (frequency, duration, sensory modality), and favorable for home use. Early pilot projects are underway to explore the feasibility of integrating light, sound, and tactile stimulation for improved entrainment and potentially multi-frequency clearance-enhancing protocols [147].

### 7.4. High-Fidelity Visualization of Drainage Routes

Novel imaging methods are revolutionizing our assessment of clearance pathways in vivo. Stereomicroscopic photoacoustic microscopy with micrometer-level resolution and millimeter depth can now simultaneously image meningeal lymphatics in parallel to parenchymal glymphatics. In mouse models of Alzheimer’s, clearance measured through meningeal lymphatics had a 70% average less clearance compared to controls, and the temporal profiles showed cate-1 peak outward efflux in the 20–40 min post administration [148]. The advantages of this technique are, firstly, the resolution, and secondly, to have a standardized specific drainage measured that can always be co-verified with MRI and multiphoton microscopy. These standardized measures can be benchmark points for computational models of solute transport, and can also be utilized in interventional studies to examine change with unmatched resolution. Additionally, it is possible to use photoacoustic reporters developed for selective detection of various amyloid-β or tau proteins to build theranostic platforms that actively track structural clearance and eventual biological/biochemically clearance in real time [149].

### 7.5. Ultrasound-Based Actuation

Ultrasound neuromodulation has emerged from systematic parameter mapping that was published in 2024. Ultrasound parameter mapping, frequency, pulse length, and duty cycle have the potential to produce uniquely different and measurable outcomes relating to neural activity, vascular tone, and perivascular pumping. Altering these parameters allows the ultrasound to be modified to exploit the slow oscillatory dynamics of clearance while staying within appropriate clinical application safety margins [150].

Ultrasound may also be uniquely combined with targeted nanocarriers to allow mechanical and local pharmacological enhancement of clearance. Ultrasound-activated stimuli-responsive nanoparticles where the payload is actively released by ultrasound stimulation have been shown to be effective in rodent models and to preferentially traffic in the perivascular space in relation to the target treatment. The ability of ultrasound to both mechanically enhance clearance and provide targeted drug delivery presents interesting possibilities for clearance approaches [151].

### 7.6. Algorithmic Acceleration of Imaging Pipelines

MRI is considered the standard clinical imaging clearance method, but traditional imaging methods have very long acquisition times and noise. Utilizing physics-aware deep learning architecture enables near super-resolution reconstruction from 4D-flow MRI scans, where errors at the vessel edges are dramatically reduced, and measuring velocity and vortex performance in the proximal perivascular spaces can be more accurate. These developments can reduce the time to complete a scan and improve landmark stabilization between machines, which is a major consideration in a multicenter clinical trial. Notably, these algorithms are not “black boxes.” They use fluid dynamics equations in the design of the algorithms to provide physical interpretability, and maintain data-driven sensitivity, which creates outputs predictable in terms of being used in a regulatory manner and thereby make good candidates for the future validation of biomarkers [152,153].

### 7.7. Digital Twin Frameworks

The digital twin concept—patient-specific computational models updated continuously with physiological data—is beginning to emerge in neurofluid research. Analogous to usage in stroke and cardiovascular medicine, clearance digital twins could merge ambulatory monitoring, imaging, and molecular biomarkers into patient-specific solute transport simulations. Initial prototypes have demonstrated that blockchain-enabled architectures can maintain data integrity and informed consent while allowing continuous updates to risk probabilities [154].

In practice, these twins could predict likely outcomes of clearance interventions prior to implementation for optimization of interventions (dosing schedules for neuromodulation, rhythm entrainment or pharmacology). It is also one route toward adaptive trials with prescriptive interventions adjusted in real time to model-predicted responses that maximize trial efficiency and reduce the burden for patients [155].

### 7.8. Environmental and Occupational Modifiers

Large studies have now shown clearance impairment as the pathway for environmental exposures to connect to neurological disease. Chronic exposure to air pollution (notable PM2.5) correlates with perivascular space dilation and small vessel disease MRI markers—likely exerting direct burden on clearance as well. While epidemiological studies of shift work point to a 25% increased risk of dementia from both sleep disruption and impaired nocturnal vasomotion, the risk of dementia among shift workers dropped back to normal if the workers had a consistent 8 h of sleep, which indicates that this factor is modifiable/protective [156]. These studies further emphasize the benefit of the conceptualization of clearance as not only a molecular target, but rather as a physiological system that is socially and environmentally modulated. Studies in which individuals are enrolled based on factoring exposure to air pollution, circadian rhythm disruption, or occupational status are likely to have a more nuanced understanding and generalizability [157].

### 7.9. Endocrine Modulation and Circadian Interventions

There is growing evidence on the role of endogenous rhythms on the regulation of clearance. In 2025, we observed consistent sleep restriction patterns; melatonin delivery yielded a subgroup pattern of AQP4 polarization. By sustaining the circadian rhythm of AQP4, immobilized sleep restriction produced clearance declines and cognitive dysfunction. Thus, a mechanistic relationship exists between circadian signaling, astrocytic polarity, and solute transport. The study led to melatonin as a low-cost adjunctive intervention and opened the door for the frontiers of circadian and hormonal modulation. Specifically, the interface for orexin, thyroid hormones, and cortisol rhythmicity has distinct implications for vascular function and astrocytic physiology; simply consider how tracking any of these interventions in relation to the availability of ongoing monitoring of clearance [158].

### 7.10. Integrated Trial Design

Collectively, the elements outlined above culminated in the outline of a translational trial design. By understanding the highest-risk participants based on inappropriate environmental exposures, occupational exposures, or imaging evidence, continuous monitoring of participants in an ambulatory model would yield a baseline of clearance physiology. The proposed interventions could be gamma entrainment, target-based ultrasound, melatonin, or a combination. Outcomes would be participant monitored via a resistance-derived device, MRI super-resolution flow imaging, and photoacoustic drainage imaging; these would triangulate outcomes to provide a measure of target engagement [159].

### 7.11. Ethical and Governance Considerations

The incorporation of continuous clearance monitoring and twin studies in prospective cohorts raises ethical considerations. There is no ownership structure for the high-resolution sleep and neural device data, and if the products are commercially or publicly innovated, it might be considered exploitation from an ownership perspective. The efficacy of therapy versus enhancement is still not a secure value: would we provide cognitive optimizing individuals, things that support clearance, besides novel products, or only in the case of disorders/disease [160].

Consensus rely/assumes interdisciplinary conversations with neuroscience and neurological researchers, ethics, regulation, and community/patient groups. The data collectives (open-science consortium), or open blockchain, consent might provide some roadmap to equitable access and informed consent. We will preemptively think about these governance issues so the technologies related to clearance could be accessibly integrated into clinical care [161].

## 8. Clinical Implications Across Neurological and Systemic Diseases

A new clinical neuro-scientific frontier is opening up with the recognition of impaired glymphatic-venous coupling as a measurable physiological disease state. That which was formerly a fantastical concept is now quantifiable in terms of clearance impairment and is of great importance to prognosis, treatment responsiveness, and surgical outcome. From the mechanisms discussed in Section 4 above such as loss of astroglial polarity, resistance to venous outflow and hypocontractility of the lymphatics, clinical studies increasingly demonstrate that failure of clearance may be regarded as a biomarker and therapeutic target in a wide spectrum of diseases.

### 8.1. Alzheimer’s Disease and Related Dementias

Large cohort population studies, including the UK BioBank study and the Rotterdam Study, indicate that those with a high burden of perivascular-space (PVS) have a 30–40% increased risk of cognitive impairment, independently of the presence of APOE4. Decreased nighttime CSF pulsatility is associated with positive amyloid-PET, suggesting that failure of glymphatic clearance is occurring upstream of amyloid deposition. Patients with less than optimal reserve capacity of clearance are found to be less favorably responsive to the anti-amyloid monoclonal antibodies and have a greater frequency of ARIA; thus, the clearance capacity may be shown to relate to a useful stratification parameter [162].

Differential diagnosis: Clearance profiling is not complete. The structural MRI markers are already in use in therapeutic trials, and the PET-tracers of clearance kinetics are at this moment being subjected to early clinical trials in humans [163].

### 8.2. Cerebrovascular Disease and Stroke

In the acute management of ischaemic stroke, the clearance-reactive 4D-flow M.R.I. detection of degree of clearance of edoema and functional improvement may be more reliable than measuring the volume of the infarct, itself. Chronic Small Vessel Disease PVS dilation and reduced diffusion-derived flux are predictors of progression of white matter hyperintensities and cognitive deterioration indicating that impaired clearance contributes independently to vascular cognitive dysfunction [164].

Readiness: Multisite prognostic trials are being performed; rehabilitation forms linked to clearance are being planned for inclusion in stroke care protocols [165].

### 8.3. Traumatic Brain Injury

Reduced nocturnal clearance subsequent to traumatic brain injury (TBI) correlates with cognitive slowing and increased predisposition to develop post traumatic epilepsy. Longitudinal imaging research in athletes and veterans shows a twofold increase in chronic disability if glymphatic transport is reduced. Over longer periods progressive failure of transport links TBI with both Alzheimer’s disease and chronic trauma encephalopathy: an echo of the mechanistic continuum described in Section 4, where repeated mechanical shear causes astroglial depolarisation and increased perivascular rigidity [166,167].

Readiness: Technological monitoring of ambulatory clearance is possible; prognostic validation is in progress [168].

### 8.4. Parkinson’s Disease and Movement Disorders

Quantitative glymphatic imaging demonstrates a flux reduction of 25–35% in Parkison’s disease, closely correlated with cognitive deterioration rather than motor deterioration. Impaired clearance is a strong predictor of dementia conversion, and assists in differentiation between idiopathic PD, atypical Parkinsonism, and prodromal REM sleep behavior disorder, allowing a meaningful diagnostic time frame [169].

Readiness: Clearance biomarkers have been validated in PD cohorts and are in progressive use for prognostication [170].

### 8.5. Multiple Sclerosis and Neuroinflammatory Disorders

Dynamic contrast-enhanced MRI shows a slowed perivascular flux around acutely demyelinating plaques and correlates positively with relapse rate and acquired disability. The patients, in whom decreased clearance is demonstrated, show less response to front-line immunomodulatory therapies, indicating that clearance monitoring would allow intensification or de-escalation of treatment [171].

Readiness: Reproducibility of imaging has been validated, whilst clearance-based therapeutic algorithms are in conceptual development [172].

### 8.6. Hydrocephalus and Idiopathic Intracranial Hypertension

In normal pressure hydrocephalus, the clearance-sensitive MRI predicts response to shunting: patients with clearance improvement postoperatively of 40% or so demonstrate the greatest improvements in gait and cognition. In IIH, improvement in drainage parasitically linked to venous sinus dilation parallels the clinical recovery phases, though in pediatric hydrocephalus, the dynamics of clearance are indices of the developmental phases of cognitive potential [173,174].

Readiness: Clearance sensitive surgical planning has commenced early pilot protocols, and there appears to be some evidence of increased prediction for outcome in early trials [175].

### 8.7. Psychiatric and Systemic Disorders

Psychiatric disorders, depression, schizophrenia, and bipolar disorder exhibit increased PVS and disrupted nocturnal clearance rhythm, which links circadian rhythm myodesopsia to the affective cognitive decline [176]. Systemic comorbidity amplifies the effect: chronic renal impairment leads to double dementia risk due to impaired clearance of solutes; heart failure leads to venous congestion, raising intracranial pressure and impaired CSF-exit; diabetes and obesity lead to increased rigidity of vascular matrices and impaired glymphatic propulsion. These observations coalesce into a cardioreno-neuro clearance axis, which extends the mechanistic triad outlined in Section 4 into systemic physiology [177].

Readiness: The epidemiologic correlation is striking, and preliminary programs are in progress towards the mapping of clearance-based screening modalities [178].

In all of these arrays of disease manifestations, the impairment of clearance emerges as a common central core of determination of outcome, bridging between neurodegeneration, vascularity, trauma, inflammation, and metabolic state. The incorporation of clearance variables within longitudinal cohorts, clinically further development of stage-specific assessment of clinical trial participation, and precision-based phase 3 recording by physicians may deliver these disparities and diverse specialisms onto a common physiological platform. It is in this circumstance of the framework that the glymphatic-venous coupling becomes a biofeedback which is capable of exploration, but also manipulation, frontier: one that is likely to breed new paradigms of recovery competence and disease-modification as they pertain to neurology and beyond [179].

Table 3 aims to give a summary of comparative disease blanks and translational readiness.

## 9. Therapeutic Frontiers and Interventional Strategies

The insight that impaired brain clearance is a dynamic, modifiable physiology, rather than a passive bystander, reframes the entire therapeutic landscape. What previously seemed to be an unmodifiable correlate of disease is now being viewed as a modifiable endophenotype, with numerous possible therapeutic targets along the glymphatic-to-venous continuum. Therapeutic strategies thus must be conceptualized not as individual levers but as a series of coordinated interventions, each corresponding to specific clearance nodes, and synchronized with physiological rhythms that regulate glymphatic egress [3,188]. The model for treatment is synergy: combinations of therapies that facilitate stabilization of astroglial polarity and motives, lowering venous congestion, and facilitating meninges lymphatic drainage, while remaining based on patient-specific endophenotypes [189]. Our high-level aim in this paper is to project the translational landscape to include pharmaceutical, device, surgical, and systemic applications.

### 9.1. Rational Combination Paradigms

Monotherapy interventions do not capture the complexity of clearance physiology, which is actively dependent upon glial architecture, vascular flow dynamics, venous compliance, and extra-craneal drainage. Combination therapies, therefore, do not represent a choice, but rather a necessity. In Alzheimer’s disease, proof-of-concept monoclonal antibodies lecanemab and donanemab glycans approved between 2023 and 2024 have established a backbone of disease-modifying therapy, though efficacy and safety are clearly mediated by clearance reserve: individuals with enlarged perivascular spaces or reductions in CSF pulsatility demonstrate diminished plaque clearance and increased risk of ARIA [190]. This allows for combination clearance interventions to be layered on top of antibody backbones. For example, applying nocturnal clearance enhancements with infusion therapies–either using sleep-promoting sounds, stabilizing ultrasound patterns, or gamma frequency sensory activation–could mitigate the risk of edema and hasten the clearance of toxic peptides [191]. In cerebrovascular disease, rational pairing of vascular pulsatility enhancers (phosphodiesterase inhibitors) and rehabilitation timed to nocturnal peaks of wedding ribwonder clearance is used to seize upon the physiological window where glymphatic transport is highest. In the venous outflow syndrome, first-line interventions may be surgical or endovascular to regain first flow, which would then rely upon pharmacological or rhythm-modulating agents to re-establish reflux. While these conventional approaches are termed ‘staged’, they aptly reflect parallels from cardiology and nephrology, and are being reframed as neuro-clearance medicine [192].

### 9.2. Chronotherapeutics and the Clearance Clock

Clearance has a rhythm, with optimal performance during consolidated slow-wave sleep. Timing clearance therapy means it is now viewed as a chronobiological prescription. Drugs with clearance-enhancing characteristics are best delivered for evening consumption to leverage drug exposure with the physiological efflux waves. Biologics might be included so that it is finished before sleep, thereby leveraging the right gradient for perivascular clearance [193].

Adaptive chronotherapy will soon be the next feasible measure, especially with wearables predicting custom sleep onset for dose adaptability. Pulse doses, even brief ones, timed to capture one or two slow-wave cycles, may be more effective than titrated steady-state exogenous delivery, to promote daytime tolerability while maximizing nighttime potential. It is important to note that non-pharmacological measures can be layered—closed-loop auditory stimulation has been shown to deepen slow waves, but would also work as a super pharmacokinetic agent that pushes solute clearance with concurrent drug delivery. In this case, the idea is not only drug clearance but drug clearance paired with a physiological brain state engagement [194].

### 9.3. Device-Based Actuation

In the past 5 years alone, there has been a tremendous growth of usable devices for clearance modification, and it has transitioned from speculation to trial readiness. Focused ultrasound (FUS) has proven to have success in oncology with transient blood–brain barrier openings, it can also produce vasomotion, and improve solute clearance. With an established safety profile as effective from glioma research, FUS has potential transit into neurodegeneration, where unique endpoints turn from tumor treatment delivery to resolving edema and improving waste clearance [195]. Gamma-band frequency sensory stimulation at 40 Hz progresses to early-stage clinical feasibility studies with the distinct aspects of light, audio, and tactile sensory stimulation. 40 Hz frequency stimulation via sensory stimulation has demonstrated improved waste clearance of amyloid and decreased waste of astroglial polarity. In humans, establishing optimal tolerability with clear cognitive endpoints of stimulation in each of the three aspects. Ultimately, endpoints using clearance mechanisms, along with cognition scales, may measure the most sensitive early clearance rate metrics [196].

Relating to the idea of “locked-in” auditory cue stimulation, combined with mismatched endogenous oscillations, creates another possible theoretical stimulation opportunity. That is not to establish an artificial rhythm, but to create and augment endogenous rhythms with a target of enhancing natural clearance peaks. Wearables and over-the-counter EEG make clearance maximized, with a patient-specific ambulatory method, clearance can seamlessly be a part of daily living and life [197].

### 9.4. Pharmacological Innovation

Pharmacological approaches are the most scalable and the most familiar way to think of intervention for clearance. Avoiding a repurposed agent approach leads to a really impressive time to translation: cilostazol, decades of off-label use tied to vascular domain thinking and presently in testing in the ability to enhance pulsatility and glymphatic clearance; SGLT2 agonists, repurposed from either diabetes or heart failure indication for vascular improvements and anti-edematous properties, with some overlap in clearance mechanisms [198]. These pragmatic agents allow for the delivery of interventions aimed at clearance in low-resource environments where technology is untenable. Along with repurposing, endocrine chronobiology is identifying new therapeutic targets. In 2025, preclinical work showed that, under sleep restriction, melatonin provided protection of AQP4 orientation and reduced clearance deficits, highlighting that circadian timing may have restorative benefits and possibly provide a mechanistic barrier as well [199]. New biological drugs may be created that either protect against astrocytic endfeet destabilization or induce meningeal lymphangiogenesis. The pharmacotherapy targets now extend to structural clearance means. These drugs will need to show site-specific action, without causing edema, and likely be introduced to humans in early dose forms in a stratified subgroup with clearance biomarkers identified [200].

### 9.5. Trial Architectures and Regulatory Pathways

A push to change the therapeutic landscape will need trial architectures commensurate with the complexity. Clearance target studies will need composite endpoints, a blend of clinical assessments, imaging, and fluid biomarkers. It may be that we create a “clearance passport,” where signals from the ALPS indices, perivascular volumetry, 4D-flow, and plasma proteomics will be combined into one endpoint. This multicomponent technique allows for reducing dependence on one modality at a time and adds an element of reproducibility [201]. Another method is the application of an adaptive trial architecture. Response-adaptive randomization, along with interim analyses based on clearances, could allow targeted recruitment of responders and reduce sham periods to interventions. Platform trials can be advantageous in that they utilize pharmacological (of all classes), devices, and balanced lifestyle therapies as a multimodal platform cross-studying infrastructure, saving time and cost [202]. Regulators are increasingly amenable to surrogate endpoints based on mechanistic rationale. Clearance enhancements could be characterized as whether safety was improved, adjuvant enhanced, or exposure was optimized, with anti-amyloid antibodies approved. This re-conceptualizing reduces evidentiary thresholds and accelerates translation [203].

### 9.6. Safety and Governance

Safety will always be the top priority in clearance therapeutics. Antibody-mediated ARIA illustrates that decreased clearance increases the risk. Pre-infusion imaging of perivascular spaces, or even venous outflow, could be used to derive an individualized assessment of risk, for potential utilization to optimize dosing or monitoring frequency. FUS protocols also need to strive towards established metrics for the acoustic dose and safety windows, as well as monitoring gamma entrainment for risk of inducing photic procession migraines or seizures. Chronotherapy carries an implicate risk of nocturnal hypotension, in that incredibly precise timing would also disrupt circadian accountable if it missed state [204].

Establishing clearance interventions into clinical pathways requires safety governance and not only efficacy endpoints. We should harmonize clearance-related adverse event reporting guidelines to generate compendium analytics, as it will ultimately enshrine regulatory learning curves [205].

### 9.7. Precision Deployment Through Endotyping

Arguably, the most revolutionary feature of the clearance therapeutics will be the re-endorsement with endotyping—defining individuals based not on a disease label, but rather on a particular form of clearance bottleneck. A pulsatility-limiting endotype could respond to treatment with vascular modulators, a venous congestive endotype would respond to stenting, an astroglia-disorganized endotype could respond to polarity stabilizers, and a lymphatic-limited endotype would be one in which the indication is based on VEGF-C therapies. This allows therapy to overlay onto the clearance overlay, instead of standardized treatment.

Newer digital twins—an individualized digitron caloric system with imaging, sleep physiology, and hemodynamics—may be utilized to model clearance response to proposed treatments, ultimately allowing us to expertise treatment regimens. N-of-1 trials, allowing patients to cycle through interventions with objective clearance metrics, are a way to advance learning and democratize optimization, especially for rare or heterogeneous clearance disorders [206].

### 9.8. Implementation, Equity, and Global Perspectives

Clearance medicine must be accessible—despite high-tech devices and biomarkers grabbing headlines, the most scalable interventions globally remain simple: sleep hygiene, blood pressure control, weight loss, CPAP adherence, and circadian regularity. These simple interventions already document measurable effects on clearance metrics and can be easily implemented worldwide. Cost-effectiveness modeling suggests that clearance stratification can decrease unnecessary exposure to expensive disease-modifying drugs and even ARIA-associated future acute hospitalizations—in an era of expensive neurotherapeutics, this will be necessary. For scenarios with low-resource limitations, a simplified clearance toolkit (1. symptom questionnaire; 2. portable handheld Doppler outflow measure; 3. wearable sleep device) offers a gradient framework to introduce clearance awareness into clinical care without advanced imaging [207,208,209].

### 9.9. Roadmap for 2025–2030

Clearing more milestones will undoubtedly solidify clearance into the medicine mainstream:

1. Regulatory qualification of composite clearance biomarker as an acceptable surrogate endpoint.
2. Adjunctive trial demonstrating clearance-timed or clearance-enhancing therapies reduce ARIA incidence or improve cognitive slope when combined with approved backbones.
3. Validated clearance endotypes, demonstrating intervention appropriately matched to clearance phenotype results in an outcome superior to standard of care.
4. Health-policy endorsement of clearance stratification as part of reimbursement criteria for expensive therapies.
5. Toolkit releases disseminated globally with low-cost clearance measures and ultimately included in dementia prevention programming initiatives.

The therapeutic area focused on the glymphatic-venous axis is poised for an important decade ahead. The most in-depth and exciting future likely lies not with singular agents but with stacked interventions—combinations of medications, devices, circadian entrainment, and global modulation that are coordinated and timed, and driven by endotype [139,210]. By including clearance biomarkers at all points of drug development, and by including therapy synchronized with circadian physiology, and high-tech and low-tech solutions at scale, the field can transition from elegant hypothesis to clinical specialty [211]. The goal is not only to clear previously abnormal clearance but also to integrate clearance as a fully equal-weighted therapeutic axis alongside metabolic, vascular, and inflammatory approaches in neurology.

## 10. Conclusions and Outlook

As this review draws to a close, we have attempted to follow the rapid development of the area of brain fluid clearance, and its incorporation into the glymphatic–venous continuum. The aim of this review is not so much to give conclusive answers to the questions raised as to bring together streams of convergent evidence from molecular, physiological, and imaging studies into an orderly whole, which may serve as a direction for future inquiry. The links between astroglial polarity, vascular pulsatility, immune regulation, and the lymphatic system have been established to present a picture that brain clearance, previously considered in a peripheral manner, is emerging as a central physiological axis, both in health and disease.

We recognize that clearing failure is not an isolated phenomenon but constitutes a broad spectrum of varying degrees of dysfunction, ranging from vascular rigidity and venous engorgement to astroglial disorganization and, ultimately, lymph clearance failure. This defect suggests the possibility that individual patients exhibiting different forms of clearing powers, endotypes, and thus gives a point of view for personalizing diagnosis and therapy. Although the data presented are largely tentative, we hope that they may serve to clarify future experimental therapies and investigations in treatment in laboratory and clinical work. From a therapeutic point of view, there are already numerous avenues of therapy through which help is beginning to appear—such as pharmacological agents reviving vascular tonus or polarity of aquaporins, devices for neuromodulation of rhythms of vasmotion or sleep, venous procedures, and systemic improvement by the use of physical agents of cardiovascular and metabolic training. These methods are not to be regarded as rivals but as complementary varieties, part of a more embracing model. By combining pharmacological, mechanical, and behavioral treatments, and synchronizing them with a circadian biological perspective, it may be possible to obtain stimulation of functional activity suited to the needs of individuals in due course.

Translationally, the nearest aspect of our point of view is to make closer the degree of closeness, or difficulty, in reaching the mechanistic level of clearing what the physical findings are. Regulatory qualification of clearance biomarkers as surrogate endpoints, of validating the physiological appearances with valid indices, clinical indices, are the unfinished tasks as yet. Plus, whether it is found that the percentage of the clearing multitude, which brings about the effects improving the clearing effect experientially occurs, increases the amount of improvement that results, or prolongs the neurodegenerative process. The advancement of these methods for progress will require, for a length of time, a close meshwork of coordination between neuroscientists and vascular or sleep physiologists, immunologists, and bioengineers.

We also wish to emphasize our perspective on equity and accessibility. While the advanced grounds of techniques such as focused ultrasound multi-omic techniques or digital twin simulations represent a terra incognita of interesting new factors, it is the more universal aspects that apply, in the sense of sleep hygiene, control of blood pressure, metabolic enhancement, and others. These universal methods are likely to have a more pronounced or spurring effect than the others mentioned above. And in this sense, the activity of the brain clearance should therefore be thought of, not only as an attitude in the nature of adjustment, but as one of the changes connected with global mental health systems. And finally, there are many points of major importance still in abeyance—on the entrance, what are the changes in the process with regard to functioning? What is the influence to be measured of the surrounding physiology as regards the behavior of man, government, behavior, neurology, and to what extent is it feasible? These troubles should not be thought too difficult, but on the contrary, a step forward will provide an opening for further study. The glymphatic–venous model is still under ’wonder’, not finished, not all of it has been processed or has gone through the correct human processes, the phenomena and effects of non-setup, interpretation lending validity to the unitary use of the query. And eventually may smoothly present to the science gathering point of empirical facts, added to by a certain degree of anxiety in hopeful pursuit of a formal growth of close points and consequences. As we present the consumer’s digest, it is not intended to be offered as fiat, but rather as an opening. A very humble piece of work, a great subject with such infinite fluidity, the working of will, and the following of the correct road. One day, we may find that the activity and dynamism in the number of the factors of evolution render the canvas of mental-associated relations surfaces infinite and accessible to no one’s mind.

## Figures and Tables

**Table 1:** Contemporary monitoring and diagnostic strategies for brain clearance. Each modality is summarized with its physiologic readout, acquisition setting, clearance-specific endpoints, validation anchors, and translational readiness. Emphasis is placed on technologies with demonstrated application in recent original human and preclinical studies.

Modality	Readout	Setting/Resolution	Clearance Endpoint	Key 2024–2025 Reference
Wireless impedance-EEG (wearable)	Parenchymal resistance during sleep	Overnight, home, ms–s resolution	Nocturnal CSF-linked impedance changes correlated with MRI tracer uptake	[88]
4D-Flow MRI Super-Resolution	CSF/venous pulsatility and vorticity	1–2 mm voxels, 8–20 min	Peak velocity, pulsatility index, flow patterns	[89]
Stereoscopic Photoacoustic Microscopy (mLV)	Meningeal lymphatic drainage dynamics	µm resolution, mm depth	Drainage volume and peak outflow timing (20–40 min)	[90]
PET–MRI dynamic tracer imaging	Tracer influx/efflux Kinetics	30–90 min scans	Inflow/outflow asymmetry, T½	[91]
Jugular Doppler Ultrasound	Venous outflow impedance	5–10 min leverages neck vessel Doppler	Velocity, collapsibility indices	[92]
Proteomic/Metabolomic Panels	Molecular clearance signatures	Days per sample	Combined biomarker signatures	[93]

**Table 2:** Interventional strategies and modulatory approaches designed to enhance glymphatic–venous clearance. Each entry specifies the target mechanism, operational parameters, chrono-timing considerations, and anticipated biomarker responses, along with key trial or cohort references.

Intervention	Target	Timing	Expected Effect	Key 2024–2025 Reference
Donanemab/Lecanemab + clearance adjunct	Amyloid removal + efflux enhancement	Infusion → nocturnal adjunct	Reduced ARIA risk, accelerated clearance	[109]
40 Hz Gamma Sensory Entrainment	Vasomotion and microglia engagement	Evening sessions	Increased perivascular transport, plaque clearance	[110]
Closed-loop SWS Stimulation	Sleep microarchitecture	Overnight auditory phase-locking	Enhanced nocturnal CSF flux	[111]
Focused Ultrasound (FUS) ± Microbubbles	Vasomotion modulation + transient BBB opening	Tuned sessions, daytime	Increased clearance and drug penetration	[112]
Venous Sinus Stenting	Outflow resistance reduction	Procedure-standard	Improved tracer efflux	[14]
CSF Shunting	Bulk CSF clearance	Shunt surgery	~40% flux increase post-op	[113]
Melatonin/Circadian Support	AQP4 polarity, rhythm stabilization	Evening dosing	Preservation of clearance rhythms	[114]
Cilostazol (PDE3 inhibitor)	Arterial pulsatility enhancement	Daily dosing	Increased glymphatic inflow	[115]
SGLT2 inhibitors (repurposed)	Vascular compliance, edema mitigation	Standard dosing	Improved vascular milieu	[116]
CPAP in OSA	Nocturnal airflow and vascular tone	Nightly adherence	Restored nocturnal clearance functions	[117]
Aerobic Exercise (structured)	Systemic vascular health	Weekly ≥150 min	ALPS index improvement (~0.1 unit)	[15]
VEGF-C Lymphangiogenesis	Meningeal lymphatic expansion	Experimental delivery	Enhanced drainage volume	[24]
CRISPR/AAV polarity repair	Astroglial structural integrity	Targeted gene therapy	Restored AQP4 localization and clearance	[41]

**Table 3:** Disease-specific interfaces of glymphatic–venous clearance failure. Biomarkers are organized by disorder, with emphasis on prognostic or decision-making leverage.

Disease/Syndrome	Biomarker	Prognostic/Decision Impact	Example Use	Reference
Alzheimer’s Disease	PVS burden, nocturnal clearance metrics	30–40% elevated risk of cognitive decline	Stratify antibody therapy; ARIA risk counseling	[180]
CSVD/Vascular Cognitive Impairment	PVS/4D-Flow pulsatility	Predict WMH growth and cognitive decline	Guide therapy intensity and follow-up	[37]
Acute Ischemic Stroke	CSF flow metrics/nocturnal clearance	Predict edema resolution and rehab response	Tailor rehab and care levels	[181]
TBI	Nocturnal clearance deficits	2× risk of long-term cognitive decline	Inform return-to-activity protocols	[182]
Parkinson’s Disease	ALPS flux decline	Dementia conversion risk stratification	Guide neurocognitive management	[183]
Multiple Sclerosis	DCE perivascular clearance near plaques	Predict relapse frequency/progression	Guide escalation of DMT	[184]
NPH/IIH	Clearance flux; venous pressure response	Anticipate surgical benefit	Select NPH shunt candidates; IIH stent sizing	[185]
Psychiatric/OSA-related	PVS, nocturnal clearance proxies	Tracks depressive or cognitive symptoms	Inform sleep/cognitive therapies	[186]
CKD/HF/Metabolic Disease	Proteomic signatures; outflow metrics	Dementia risk stratification	Scaffold preventive strategies	[187]

## Data Availability

The data presented in this study are available on request from the corresponding author.
